# Enabling efficient and robust analysis of tandem repeats in genomic data using Wavefront-based String Decomposer

**DOI:** 10.1101/gr.281346.125

**Published:** 2026-06

**Authors:** Junhai Qi, Zhidong Yang, Ting Yu, Guojun Li

**Affiliations:** 1 Research Center for Mathematics and Interdisciplinary Sciences (Frontiers Science Center for Nonlinear Expectations), Shandong University, Qingdao, Shandong 266237, China;; 2 Department of Chemical and Biological Engineering, The Hong Kong University of Science and Technology, Hong Kong S.A.R. 999077, China

## Abstract

Tandem repeat (TR) analysis is crucial for understanding genome structure and variation. However, string decomposition, a key challenge in TRs analysis, remains computationally demanding. In this study, we introduce Wavefront-based String Decomposer (WSD), a novel algorithm that enhances efficiency and accuracy in TRs decomposition. By integrating wavefront techniques, WSD significantly reduces computational and memory costs. Additionally, two adaptive strategies minimize parameter sensitivity and further improve efficiency. Through extensive experiments, we demonstrate that WSD outperforms current state-of-the-art (SOTA) methods, achieving an average speedup of ∼2.33× and reducing memory usage by two orders of magnitude when analyzing human TRs.

TRs are genomic sequences composed of tandemly arranged DNA repeat units. TRs are broadly categorized into short tandem repeats (STRs) and long tandem repeats (LTRs) based on repeat unit length. STRs typically consist of 1–6 base pair (bp) repeat units, whereas LTRs can span hundreds of base pairs. In human centromeric *α*-satellite regions, the core repeat unit—the monomer—is approximately 171 bp in length. Monomers can further organize into higher-order repeats (HORs), which are repeated hundreds to thousands of times. STR expansions contribute to approximately 60 human genetic disorders (Depienne and Mandel 2021), whereas LTRs are associated with diseases such as pancreatic cancer (Ting et al. 2011).

A key computational challenge in TR analysis is the string decomposition problem (decomposing TRs into repetitive units). Dvorkina et al. (2020) formally defined this problem and introduced StringDecomposer (SD), a computational method that models decomposition as a longest-path search in a string decomposition graph. Despite advancements, complete centromere assembly remains challenging due to the complexity of repetitive structures (Li and Durbin 2024). Leveraging SD, CentroFlye (Bzikadze and Pevzner 2020) was developed as a centromere-specific assembly tool, enabling the first complete reconstruction of human Chromosome 6 and X. Furthermore, string decomposition algorithms play a critical role in resolving mosaic tandem repeats (Masutani et al. 2023) and HORs (Kunyavskaya et al. 2022; Gao et al. 2023; Qi et al. 2025), facilitating large-scale comparative analyses of TR variation across human populations (Ichikawa et al. 2023; Gao et al. 2024; Logsdon et al. 2024).

The string decomposition problem is fundamentally a dynamic programming (DP) problem (see Methods section). Although analogous to sequence alignment, it exhibits higher computational complexity in both time and space, making traditional DP-based methods impractical for large-scale genomic data sets. Most sequence alignment algorithms rely on classical Needleman-Wunsch-DP paradigms (Needleman and Wunsch 1970). However, recent advancements in wavefront-based approaches led to the development of wavefront alignment (WFA) (Marco-Sola et al. 2021), which greatly improves efficiency in the global alignment of highly similar sequences. Several modern alignment tools now incorporate WFA algorithm to optimize performance (Bahk and Sung 2024; Zhang et al. 2025).

In this study, we introduce the wavefront paradigm and present Wavefront-based String Decomposer (WSD), a new algorithm for fast string decomposition. By incorporating two adaptive strategies, WSD achieves markedly higher speed and lower memory consumption, while reducing the sensitivity of decomposition results to parameter settings. These improvements enable more robust, accurate, and efficient analysis of TR reads and assemblies.

## Results

### Overview of WSD

**Figure 1.**
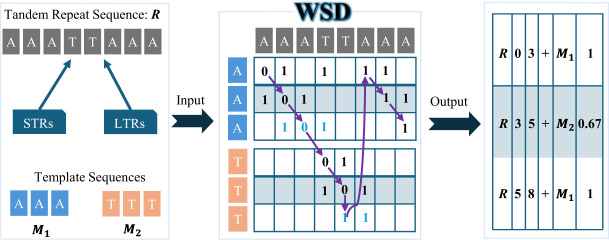
Overview of WSD. WSD constructs a DP matrix similar to sequence alignment (with mismatch and gap penalties set to 1 in this example). Each cell in the matrix represents the minimum decomposition cost at that position. The purple arrows indicate the decomposition path, analogous to an alignment path, which determines the final decomposition result. Blue-labeled DP cells represent “template-transition” cells, allowing the decomposition path to jump between specific DP cells of different templates. The WSD output is a TSV file with columns: TR name/TR start/TR end/alignment direction (“+” for forward, “−” for reverse complement)/template name/identity score.

Figure 1 illustrates the workflow of the WSD algorithm. The input to the WSD algorithm consists of TRs and a set of template sequences, which can be either STRs or LTRs, while the output is a decomposition result. The decomposition comprises an ordered set of nonoverlapping blocks, each representing a subsequence of the tandem repeat sequence with a defined start and end position. Each block is associated with a specific template sequence and an identity score, indicating the degree of similarity between the block and the template.

Fundamentally, WSD is a DP-based algorithm, primarily composed of the extend and expand functions (details in Methods section). Given a fixed cost score, the extend function attempts to extend along diagonals in the DP matrix that satisfy specific conditions until reaching the final cell. If no diagonal can reach the final cell due to an insufficient cost score, the expand function activates, enabling additional diagonals to extend. This iterative process continues until a diagonal successfully reaches the final cell.

To enhance computational efficiency, WSD employs an adaptive partitioning strategy, segmenting the tandem repeat sequence into fixed-length subsequences, individually decomposing them, and merging the results to obtain the final decomposition. Additionally, an adaptive pruning strategy is integrated to further accelerate the iterative process.

### Benchmarking of WSD

There are many tools for tandem repeat analysis, such as TRF (Benson 1999) and NCRF (Harris et al. 2019), but none of them explicitly solve the string decomposition problem, which has been extensively discussed (Dvorkina et al. 2020). TRviz (Park et al. 2023) was developed for decomposing and visualizing tandem repeat units, whereas vamos (Ren et al. 2023) focuses on annotating variable-number tandem repeats (VNTRs). A common subproblem in these methods is string decomposition. However, their decomposition approaches are fundamentally similar to SD (vamos uses SD directly); SD currently represents SOTA. Therefore, WSD was benchmarked exclusively against SD.

We compared WSD and SD on both simulated and real data sets. The experimental environment and command lines are provided in Supplemental Note 1. The simulated data sets include simulated assembly data sets and simulated sequencing data sets. The simulated assembly data sets were generated following the methodology of HiCAT (Gao et al. 2023), with the same simulation script parameters, resulting in 36 different types of data sets (S1–S36, see Supplemental Table S1), each containing 100 assemblies. The simulated sequencing data sets have different read lengths and error rates (SR1–SR24, see Supplemental Table S2), each data set contains 500 reads. The generation details are provided in Supplemental Note 2. On the simulated data set, two decomposition methods can be applied: monomer decomposition (using monomers as templates to complete the decomposition) and HOR decomposition (using HORs as templates to complete the decomposition).

Additionally, we evaluated the algorithms on well-characterized centromeric regions from two reference genomes: the centromeres of the human CHM13-T2T genome (v2.0) (Nurk et al. 2022) and those of *Arabidopsis thaliana* (Naish et al. 2021). Given the heterogeneity of HOR organization in real centromeres, and the fact that HORs do not necessarily share a fixed number or ordering of monomers, we performed decomposition on these real data sets using monomers as the fundamental templates. This choice allows a consistent and general evaluation of algorithmic performance under realistic centromeric complexity. Centromeric region templates were obtained from HORmon (Kunyavskaya et al. 2022).

The primary evaluation metrics are the running time and memory usage. Theoretically, both algorithms are exact and should guarantee perfect decomposition across all data sets. However, to accelerate computation, TRs are often divided into a series of overlapping subsequences. The decomposition results of these subsequences are then merged to obtain the final decomposition of TRs. This approach may miss the optimal decomposition, so we also introduced three metrics to evaluate accuracy: bias, decomposition accuracy, and decomposition distance, defined as follows.

Both methods produce decompositions as a series of contiguous blocks, where each block has a start position and an end position on the reference, a template label, and an identity score representing its similarity to the template. We define *GTN* as the ground-truth number of blocks and *AN* as the number of blocks obtained by the algorithm. Bias is calculated as

(1) Bias=|GTN−AN|.

When the bias is 0, assuming the true template label of the *i*th block is *TM*_*i*_ and the template label obtained by the algorithm is *AM*_*i*_, and the decomposition accuracy is defined as

(2) $$\text{Accuracy}=\frac{\left|\{AM_{i}|\text{if}\;AM_{i}=TM_{i},\;i=1,2,\ldots ,GTN\}\right|}{GTN}.$$

Because no ground-truth template labels are available for the real data set, we cannot directly compute decomposition accuracy. Therefore, we estimate the algorithm’s accuracy on real data using the decomposition distance and decomposition identity. Let $S_{AM_{i}}$ denote the sequence corresponding to the template labeled *AM*_*i*_, and let *R* be the input tandem repeat sequence. The reconstructed sequence *RS* is denoted as $S_{AM_{1}}\circ S_{AM_{2}}\circ \cdots \circ S_{AM_{AN}}$, where the symbol “∘” represents the concatenation operation. The edit distance between *RS* and *R* is defined as the decomposition distance:

(3) Decomposition distance=Edit Distance(R,RS).

Correspondingly, the identity between *RS* and *R* is then defined as the decomposition identity:

(4) $$\text{Decomposition identity}=1-\frac{\mathrm{Edit}\;\mathrm{Distance}(R,RS)}{\mathrm{Length}(R)}.$$

### WSD outperforms SD on simulated data sets

**Figure 2.**
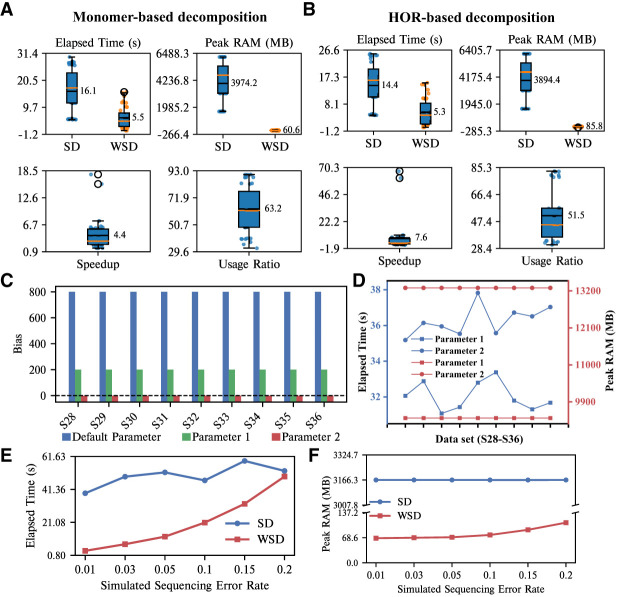
Evaluation of the two methods on simulated data sets. (*A*) Runtime and peak memory usage for the decomposition of R1–R36 using monomers as templates, along with the speedup and usage ratio of WSD. (*B*) Runtime and peak memory usage for the decomposition of R1–R36 using a HOR as a template, along with the speedup and usage ratio of WSD. (*C*) Bias of SD with three different parameter settings using a HOR as a template. Parameter 1: *stringDecomposer -b 7500 -v 1000*. Parameter 2: *stringDecomposer -b 10,000 -v 2000*. (*D*) Running time and memory usage of SD with two different parameters. (*E*) Runtime of all methods on data sets (SR19-SR24) with different sequencing error rates. (*F*) Peak memory usage of all methods on data sets (SR19-SR24) with different sequencing error rates.

We first compared WSD and SD on simulated assembly data sets, and the specific results are shown in Figure 2. Whether the string decomposition is based on monomers or HORs, WSD demonstrates clear advantages in both speed and memory usage, speedup greater than 4.4 and usage ratio greater than 51.5 (see Fig. 2A,B). WSD can complete the decomposition of a data set in 0.08–15.81 s, requiring only 20–126 MB of memory, whereas SD takes 4.3–29.89 s and requires 1558–6182 MB of memory. Exact values can be found in Supplemental Tables S3 and S4. Specifically, when decomposing sequences based on monomers, WSD is up to 17 times faster than SD, using only 1/40 of the memory. When decomposing sequences based on HORs, WSD is up to 53 times faster, requiring only 1/82 of the memory of SD.

Additionally, Supplemental Table S5 shows that when decomposing sequences based on monomers, both methods achieve perfect decomposition results across data sets S1–S36. However, when decomposing sequences based on HORs, SD shows significant bias on data sets S10–S36, and WSD still achieves perfect decomposition results (see Supplemental Table S6).

We observe that SD has lower decomposition accuracy and higher bias on data sets with longer templates. To address this, we re-ran SD on data sets S28–S36 with two different parameter settings, and obtained new results. As shown in Figure 2C, compared to the results using the default parameters, SD’s bias significantly reduced under parameters 1 and 2, with parameter 2 achieving a bias of 0 (accuracy is also 100%). These parameters determine how the TR is divided into subsequences, and different divisions yield varying decomposition accuracies. Although SD also achieved the perfect decomposition result with parameter 2, it also led to longer running times and increased memory usage (Fig. 2D). In contrast, WSD adopts a simple strategy that adaptively decides a subsequence division based on template length (see Methods section), ensuring the robust decomposition results.

The performance of WSD depends on the divergence between the template and the target tandem repeat sequences (see Methods section). To assess this, we evaluated WSD and SD on simulated sequencing data sets (SR1–SR24) generated with different error rates, where higher error rates indicate greater divergence between the template and the sequences. Error rates of 0.01, 0.03, and 0.05 represent typical modern sequencing conditions, whereas error rates of 0.10, 0.15, and 0.20 represent low-quality sequencing. Across all data sets SR1–SR24, both WSD and SD consistently produced correct decompositions (Supplemental Tables S7 and S8). In terms of computational efficiency and memory usage, WSD showed a clear advantage. For example, at an error rate of 0.01 with a read length of 5000 bp (SR1), WSD completed the decomposition of SR1 in 2 s, which was about 30 times faster than SD, while using only 1/59th of the memory used by SD. With a read length of 20,000 bp, WSD remained about 10 times faster than SD and used only 1/47th of the memory. Detailed results can be found in Supplemental Tables S9 and S10. As the read length increased to 20,000 bp and the error rate became higher, the performance difference between WSD and SD gradually decreased (Fig. 2E). When the sequencing error rate reached 0.20, WSD and SD showed similar runtime performance; however, WSD continued to show a clear memory advantage (Fig. 2F).

### WSD outperforms SD on real-world data sets

We compared WSD and SD on human centromeric assemblies and Arabidopsis centromeric assemblies. In terms of decomposition results, WSD and SD produced nearly identical outputs, with small differences in the number of decomposed blocks, decomposition distance, and the decomposition identity. Exact values can be found in Supplemental Tables S11 and S12, and Tables 1 and 2.

**Table 1.** Performance of the two methods on human centromeric assemblies using 36 threads

Chromosome	Elapsed time (s)		Speedup	Peak RAM (MB)		Usage ratio	Decomposition identity	
	SD	WSD		SD	WSD		SD	WSD
1	500.26	237.03	2.11	138,271.14	2379.06	58.12	0.9878	0.9879
2	370.17	281.42	1.32	138,263.84	1774.79	77.90	0.9801	0.9801
3	307.10	98.37	3.12	138,259.23	412.61	335.09	0.9924	0.9924
4	283.96	102.70	2.76	138,258.62	269.03	513.92	0.9905	0.9905
5	395.54	203.87	1.94	138,264.43	1353.27	102.17	0.9869	0.9869
6	412.96	89.49	4.61	138,265.18	1651.03	83.74	0.9963	0.9963
7	391.33	150.76	2.60	138,266.92	1703.16	81.18	0.9936	0.9936
8	349.36	400.03	0.87	138,263.24	1586.27	87.16	0.9912	0.9912
9	355.95	162.37	2.19	138,264.88	1261.57	109.60	0.9854	0.9854
10	355.01	152.76	2.32	138,263.00	907.48	152.36	0.9875	0.9875
11	434.39	137.02	3.17	138,267.09	1700.92	81.29	0.9866	0.9866
12	391.67	191.91	2.04	138,265.51	1228.88	112.51	0.9877	0.9877
13	362.69	77.61	4.67	138,262.50	1022.98	135.16	0.9949	0.9949
14	377.61	111.55	3.39	138,265.47	1469.97	94.06	0.9903	0.9902
15	293.88	115.67	2.54	138,259.65	500.17	276.42	0.9908	0.9908
16	363.92	373.82	0.97	138,262.79	964.52	143.35	0.9889	0.9889
17	448.27	505.54	0.89	138,268.64	1806.00	76.56	0.9921	0.9920
18	476.73	220.09	2.17	138,271.41	2470.22	55.98	0.9885	0.9885
19	450.41	215.36	2.09	138,268.91	2147.89	64.37	0.9833	0.9833
20	367.74	116.95	3.14	138,263.62	980.42	141.02	0.9909	0.9909
21	251.38	154.96	1.62	134,888.57	161.95	832.93	0.9859	0.9860
22	421.47	173.22	2.43	138,266.49	1211.66	114.11	0.9902	0.9902
X	440.30	531.44	0.83	138,266.33	1712.48	80.74	0.9894	0.9893

SD uses the default parameters, whereas WSD uses the parameter *“-A1”* with all other parameters set to their default values.

**Table 2.** Performance of the two methods on *Arabidopsis* centromeric assemblies using 36 threads

Chromosome	Elapsed time (s)		Speedup	Peak RAM (MB)		Usage ratio	Decomposition identity	
	SD	WSD		SD	WSD		SD	WSD
1	14.97	10.41	1.44	5593.40	35.07	159.51	0.9682	0.9681
2	12.52	7.97	1.57	5592.08	32.54	171.86	0.9649	0.9650
3	11.60	7.47	1.55	5591.11	29.01	192.75	0.9585	0.9586
4	12.89	9.68	1.33	5592.02	34.76	160.89	0.9547	0.9548
5	9.86	10.99	0.90	5590.27	22.68	246.49	0.9524	0.9521

SD uses the default parameters, whereas WSD uses the parameter *“-A1”* with all other parameters set to their default values.

WSD demonstrates significant advantages in both speed and memory usage. As shown in Tables 1 and 2, WSD outperforms SD in decomposition speed for the majority of centromeres, with only a few cases where it is slightly slower. Notably, all of these slower cases share a common feature: they contain extremely long high-divergence regions exceeding 4400 bp (see Supplemental Fig. S1). Compared to human centromeres, both methods can decompose Arabidopsis centromeres more efficiently due to their shorter length and significantly lower number of monomers. Notably, when decomposing the centromere of human Chromosome 13, SD takes 362.69 s, whereas WSD completes the task in just 77.61 s—a 4.67-fold speedup. On average, WSD is 2.33 times faster across human centromeres.

In addition to speed, WSD also has a substantial memory advantage. When processing human centromeres, SD requires at least 138,300 MB of memory, whereas WSD needs only 2500 MB. On average, WSD’s memory consumption is only 1/165 that of SD. Similarly, when analyzing Arabidopsis centromeres, WSD maintains its memory efficiency, requiring only 40 MB, whereas SD needs at least 5594 MB. On average, WSD uses just 1/186 of the memory required by SD.

## Discussion

In this study, we developed a novel sequence decomposition algorithm called WSD to enable more accurate, stable, and efficient TRs analysis. By leveraging the wavefront technique, WSD significantly reduces the computational and memory costs associated with sequence decomposition. Additionally, the incorporation of two adaptive sequence partitioning/pruning strategies mitigates parameter sensitivity and further enhances computational efficiency. Simultaneously, restricting penalties to integers simplifies dynamic programming and ensures numerical stability without compromising qualitative behavior. Collectively, this optimized efficiency unlocks the potential for population-level studies of alpha satellite regions, which involves processing thousands of genomes. Furthermore, our method facilitates the integration of these complex regions into pangenome graphs, a task that requires both accuracy and computational efficiency.

Although extensive tests show that WSD efficiently decomposes both TR assemblies and long reads, its computational performance is influenced by the divergence between the input sequence and the template set. When the divergence becomes large (e.g., above 20%), the speed advantage of the algorithm may decrease. In practical situations, regions with high divergence, such as pericentromeric segments, leads to slower computation. These findings indicate that WSD is most effective when it is applied to genomic regions that have moderate or high similarity to the templates.

Based on these characteristics, several directions may help further improve WSD. One possible direction is to add preprocessing modules that detect regions that do not contain tandem repeats or regions that show weak similarity to the template set. Limiting WSD to intervals with strong similarity would allow the algorithm to maintain its computational advantages and reduce unnecessary processing in low-similarity regions. Building on this idea, a hybrid framework that estimates local divergence and selectively applies either WSD or a standard alignment-based method could further improve efficiency while preserving the advantages of WSD in regions where repeat structure is well maintained. In addition, incorporating automatic identification of tandem array boundaries would improve usability and facilitate large-scale analyses, representing an important direction for future development.

Memory usage is another area where WSD may be improved. Incorporating ideas from biWFA (Marco-Sola et al. 2023) may help WSD reduce complexity. In addition, the current implementation uses a linear-gap scoring model for the sake of efficiency. Adding support for an affine-gap scoring model would allow WSD to better handle cases that involve structural insertions, such as retrotransposons. Conceptually, extending WSD to affine-gap scoring is possible, but it requires modifying the dynamic-programming formulation. In particular, each block alignment would need to maintain three wavefront states, match or mismatch (M), insertion (I), and deletion (D), as in the classical affine-gap model (Gotoh 1982). In a wavefront framework, this means that three related wavefronts must be computed along each diagonal, with each wavefront following its own recurrence and extension rules. The template-transition mechanism would also need to propagate these states correctly across template boundaries, whereas the extension procedure would continue to operate only on the M state.

It is important to note that the completeness of sequence decomposition depends on the completeness of the input monomer library. This is a general property of methods that rely on predefined monomer sets, including WSD and StringDecomposer. When real centromeres contain highly divergent monomers or novel variants that are not represented in the input library, sequence similarity decreases and these regions may not be included in the reconstructed sequence. As a result, aligning the reconstructed sequence back to the genome can reveal large gaps, often with lengths that are multiples of the monomer size, corresponding to these unrepresented variants. Therefore, improving and expanding the monomer library to better capture centromere sequence diversity is an important direction for reducing such gaps in future analyses.

## Methods

### String decomposition problem

Let *R* = *r*_1_ *r*_2_ · · · *r*_*n*_ be a tandem repeat sequence, and let *M*_1_, *M*_2_, …, *M*_*t*_ be a series of template sequences, where $M_{j}=m_{\;j1}m_{\;j2}\cdots m_{\;jl_{j}}$. Let block *B*_*α*_ be a subsequence of *R*, and let interval(*B*_*α*_) denote the coordinate interval—consisting of the start and end positions of *B*_*α*_ on *R*. Then, the string decomposition problem can be formulated as the following optimization problem:

(5) $$\begin{array}{rl} \underset{1\leq x\leq n}{\min} & \;\sum_{\alpha =1}^{x}\;\mathrm{cost}(B_{\alpha },M_{{\text{β}}_{x}}),\;{\text{β}}_{x}\in \{1,2,\ldots ,t\},\\ \text{s.t.} & \;\left\{ \begin{array}{c} B_{1}\circ B_{2}\cdots \circ B_{x}=R,\;\\ \forall \;1\leq \gamma ,\delta \leq x,\;\gamma \neq \delta ,\;\mathrm{interval}(B_{\gamma })\cap \mathrm{interval}(B_{\delta })=\emptyset .\; \end{array}\right. \end{array}$$

Here, the function cost(∗) represents the cost of aligning two strings. Next, we demonstrate how to solve the above optimization problem based on the WSD algorithm.

### A sequence-alignment-like DP solution

SD introduces the construction of a string decomposition graph (SD graph), where the longest (heaviest) path in the graph represents the optimal solution to the string decomposition problem (Dvorkina et al. 2020). In fact, the heaviest path in the SD graph corresponds directly to the optimal solution of Equation 5. If the decomposition represented by a path in the SD graph consists of *x* blocks, then the path contains *x* − 1 “block-switching” edges (each with weight 0). The total weight of this path is therefore the sum of the weights contributed by the *x* alignment graphs associated with these blocks. Note that although the edge weights in the SD graph are formulated so that a larger weight corresponds to a better (i.e., lower-cost) block alignment, the weight assigned to each block is exactly a monotonic transformation of the cost defined in Equation 5. Consequently, maximizing the path weight in the SD graph is equivalent to minimizing the total cost in Equation 5. Hence, the optimization problem in Equation 5 is mathematically equivalent to finding the heaviest path in the SD graph.

String decomposition can be formulated as a DP problem analogous to sequence alignment. Specifically, assume that the penalty score for a mismatch is *h* and the penalty score for a gap is *g*, where both *h* and *g* are strictly greater than 0. When character *y* is not equal to *z*, the penalty function is defined as *s*(*y*, *z*) = *h*; otherwise, it is 0. We define a three-dimensional DP matrix *D*, where *i*, *j*, *k* are the indices corresponding to the input sequence *R*, the template set, and the template sequence within the set, respectively. The cell *D*[*i*][*j*][*k*] represents the optimal decomposition cost of the prefix subsequence *R*′ = *r*_1_ *r*_2_ · · · *r*_*i*_ (*i* ≤ *n*) under the specific condition that the decomposition ends with the block corresponding to the subsequence *m*_*j*1_ *m*_*j*2_ · · · *m*_*jk*_ of the *j*th template. Thus, each entry *D*[*i*][*j*][*k*] encodes the best cost among all decompositions of *R*′ that terminate in this particular template-state configuration. To obtain the overall optimal decomposition cost of *R*′, we take the minimum over all templates *j* and valid template positions *k*:

(6) $${\mathrm{Cost}}_{i}=\min_{1\leq i\leq n,\;1\leq j\leq t,\;1\leq k\leq l_{j}}D[i][j][k],$$

where *t* is the number of templates and *l*_*j*_ represents the length of the *j*th template. Similar to sequence alignment, when *i* = 1 and *k* = 1, for all *j*, we have *D*[1][*j*][1] = *s*(*r*_1_, *m*_*j*1_). When *i* = 1 and *k* > 1, we have

(7) $$D[1][j][k]=\min\left((k-1)\cdot g+s(r_{1},m_{\;jk}),\;D[1][j][k-1]+g\right).$$

When *i* > 1, we have the following recurrence relation:

(8) $$D[i][j][k]=\min\left\{ \begin{array}{cc} D[i-1][j][k-1]+s(r_{i},m_{\;jk})\; & (k>1),\\ D[i][j][k-1]+g\; & (k>1),\\ D[i-1][j][k]+g\; & (k\geq 1),\\ \min_{1\leq \;j_{*}\leq t}D[i-1][\;j_{*}][l_{\;j_{*}}]+s(r_{i},m_{\;jk})\; & (k=1). \end{array}\right.$$

We can regard *D* as *t* sequence alignment DP matrices corresponding to *R* and each *M*_*j*_. Unlike a standard sequence alignment DP matrix, when *k* = 1, *D*[*i*][*j*][*k*] depends not only on its left neighbor *D*[*i* − 1][*j*][*k*] but also on all $D[i-1][\;j_{*}]{\left[l_{\;j_{*}}\right]}_{1\leq \;j_{*}\leq t}$. This additional dependency reveals a template-transition in the string decomposition problem—namely, once the sequence alignment reaches the last character of a particular template, the subsequent alignment step may jump to the first character of another template.

Once *D* is computed, similar to sequence alignment, we start backtracking from a cell with cost $\min_{1\leq j\leq t,\;1\leq k\leq l_{\;j}}D[n][j][k]$ in *D* until reaching another cell *D*[1][*j*][1], 1 ≤ *j* ≤ *t*. The backtracking path records the decomposition result of *R*. The computational complexity and space complexity of this DP scheme are both $O(n\cdot \sum_{\;j=1}^{t}l_{j})$. Although this DP approach is elegant, its runtime and memory consumption increase significantly when *R* is long and the template set is large. The WSD algorithm introduces a novel DP strategy that eliminates the need to compute and store many DP cells while still obtaining the decomposition results. Details are provided below.

### WSD algorithm: a wavefront-based DP solution

**Figure 3.**
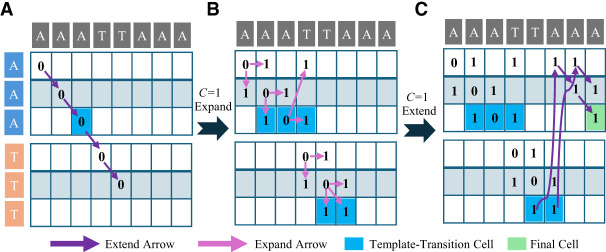
A computational example of WSD (with mismatch and gap penalties set to 1 in this example). Arrows of different colors indicate the origin of the cell. (*A*) When the cost score *c* = 0, the extend function is executed. (*B*) When *c* = 0 and the final cell cannot be reached, the cost score *c* is incremented and the expand function is executed. (*C*) When the cost score *c* = 1, the extend function is executed.

Figure 3, parts A–C, shows a computational example of WSD. “Wavefront” is essentially a set of cells in the DP matrix that satisfy certain conditions (Marco-Sola et al. 2021). Specifically, we consider the matrix *D* and a specific cell (*i*, *j*, *k*) within it, where *i* and *k* correspond to the indices of the reference sequence *R* and the template sequence *M*_*j*_, respectively, and we define the position of this cell in the sequence alignment-like DP matrix for the *j*th template. Specifically, we denote this position in a diagonal with index *d*, where *d* = *i* − *k*, and represent this diagonal as an ordered pair (*j*, *d*).

Given a cost score *c*, we define *F*_*c*,*j*,*d*_ as the farthest cell along the diagonal (*j*, *d*) that maintains a cost score of *c*. The reference index of *F*_*c*,*j*,*d*_, referred to as the *offset*, is denoted by *W*_*c*,*j*,*d*_. A cell Cell_*x*_ is considered farther than a cell Cell_*y*_ along the same diagonal if its *offset* is greater than that of Cell_*y*_. We define a function ETP (*E*xtending *T*he *P*refix):

(9) ETP(W,R,M):=W+LCP(R[W+1:],M[W+1−d:]).

Similar to Equation 8, the recursive relation for *W*_*c*,*j*,*d*_ can be derived as follows:

(10) $$W_{c,j,d}=\max\left\{ \begin{array}{c} \mathrm{ETP}(W_{c-h,j,d}+1,R,M_{\;j}),\;\\ \mathrm{ETP}(W_{c-g,j,d+1},R,M_{\;j}),\;\\ \mathrm{ETP}(W_{c-g,j,d-1}+1,R,M_{\;j}),\;\\ \mathrm{ETP}(d+1,R,M_{\;j})\;(\text{if}\;\exists j^{\prime }\;(1\leq j^{\prime }\leq t),\;s.t.\;W_{c-h,j^{\prime },d-l_{\;j^{\prime }}}\\ =d\;\&\&\;d\geq 0). \end{array}\right.$$

Here, LCP(*, *) denotes the length of the longest common prefix between two strings, and the notation [:] represents the slicing operation. For example, given an integer *p*_*x*_ (1 ≤ *p*_*x*_ ≤ *n*), *R*[*p*_*x*_ :] represents the subsequence $r_{\;p_{x}}r_{\;p_{x}+1}\cdots r_{n}$. Initially, for each *j*, if *r*_1_ = *m*_*j*1_ the variable *W*_0,*j*,0_ is initialized to 1; otherwise, it is initialized to 0. Supplemental Figure S2 further visualizes the dependencies of Equation 10.

Algorithm 1: **WSD algorithm**

  **input**: Reference sequence *R* = *r*_1_*r*_2_ · · · *r*_*n*_, template set $T\leftarrow \{M_{1},M_{2},\ldots ,M_{t}\}$, gap/mismatch penalty score *g*/*h*

** output**: String decomposition result $\tilde{D}$

1 *c* ← 0; offset ← 0; *  W*_0,*j*,0_ ← 1 (if *r*_1_ = *m*_*j*1_) or 0 (otherwise)   ▹ Initial conditions

2 **while** *true* **do**

3  extend(*R*, *T*, *W*, offset, *c*) ▹ Calculate Part 1

4  **if** offset ≥ *n* **then**

5    break▹ Decomposition is finished

6  *c* ← *c* + 1

7  expand(*W*, *c*)▹ Calculate Part 2

8 $\tilde{D}\leftarrow \mathrm{Backtrace}(W,c,g,h,R,T)$

9 **return** $\tilde{D}$

The WSD algorithm is centered around Equation 10 (see Algorithm 1). To facilitate understanding, each recurrence relation in Equation 10 can be divided into two components: the first component is the LCP function (referred to as Part 1), and the second consists of the remaining terms in the equation (referred to as Part 2). Part 1 is computed using the extend function (see Algorithm 2), whereas Part 2 is handled by the expand function (see Algorithm 3). There is a subtle but important detail here. Algorithm 3 suggests that we first determine the maximum among *W*_*c*−*h*,*j*,*d*_ + 1, *W*_*c*−*g*,*j*,*d*+1_, and *W*_*c*−*g*,*j*,*d*−1_ + 1, and then perform the extend procedure starting from this maximum value. In contrast, Equation 10 implies that the extend procedure should be executed starting from all three values *W*_*c*−*h*,*j*,*d*_ + 1, *W*_*c*−*g*,*j*,*d*+1_, and *W*_*c*−*g*,*j*,*d*−1_ + 1, followed by taking the maximum of their extension results. We claim that these two approaches are equivalent; that is: ETP(max(a,b), R, M)=max(ETP(a, R, M),ETP(b, R, M)).

The details of the proof are in Supplemental Note 3.

Algorithm 2: **Extend function**

 **input** : Reference sequence *R* = *r*_1_*r*_2_ · · · *r*_*n*_, template set $T\leftarrow \{M_{1},M_{2},\ldots ,M_{t}\}$, wavefront *W*, offset offset, cost score *c*

 1 Function: ExtendDiagonal(*W*, offset, *u*, *v*, *j*, *d*)

 2  **while** *r*_*u*_ = = *m*_*jv*_ **do**

 3   $W_{c,j,d}\leftarrow W_{c,j,d}+1$

 4   *u* ← *u* + 1

 5   *v* ← *v* + 1

 6  **if** $W_{c,j,d}==\min(v+l_{j},n)$ **then**

 7    *q*.*push*([*j*, *d*])▹ Obtain a template-transition cell

 8  $\mathrm{offset}=\max(\mathrm{offset},W_{c,j,d})$

 9  **if** offset ≥ *n* **then**

10   **return**

11 Extend(*R*, *T*, *W*, offset, *c*)

12  *q* ← queue()▹ A FIFO queue to store template-transition cells

13  **for** 1 ← *j* to *t* **do**

14    for 1 − *l*_*j*_ ← *d* to *n* − 1 **do**

15      **if** *W*_*c*,*j*,*d*_ ≠ 0 **then**

16       $u\leftarrow W_{c,j,d}+1$

17       $v\leftarrow W_{c,j,d}-d+1$

18       ExtendDiagonal (*W*, offset, *u*, *v*, *j*, *d*)

19  **while** *q not empty* **do**

20    [x,y]←q.pop()

21    $p_{r}\leftarrow W_{c,x,y}$

22    **for** 1 ← *j* to *t* **do**

23      **if** $r_{\;p_{r}+1}==m_{\;j1}$ **then**

24       $W_{c,j,p_{r}}\leftarrow \;p_{r}+1$

25       $u\leftarrow W_{c,j,p_{r}}+1$

26       *v* ← 2

27       ExtendDiagonal (*W*, offset, *u*, *v*, *j*, *p*_*r*_)

28      **else**

29        **if** $W_{c+h,j,p_{r}}==0$ **then**

30         $W_{c+h,j,p_{r}}\leftarrow p_{r}+1$▹ This suggests a mismatch so expand $W_{c,j,p_{r}}$ to obtain $W_{c+h,j,p_{r}}$

The extend function iteratively verifies whether characters at corresponding positions along each diagonal are identical. When a mismatch occurs, it signifies that the farthest point under the current cost *c* has been reached. If the offset at this point remains less than *n*, it indicates that the minimum decomposition cost of *R* exceeds *c*. In this case, *c* is incremented, and the expand function is executed. The expand function initializes a new wavefront for selected diagonals under the updated cost *c* + 1. The extend function then utilizes this newly established wavefront to continue extending the diagonals until their respective farthest points are reached. Notably, the extend function consists of two steps. First, it operates within the DP matrix of the corresponding template (Algorithm 2, lines 11–18), adding all template-transition cells to a queue *q*. Then, the extension proceeds based on *q* (Algorithm 2, lines 19–30): although the current alignment may reach the end of a block on a particular template, the match does not necessarily terminate at this cell. It is still possible to continue extending the decomposition by switching to other templates. When the offset is greater than or equal to *n*, backtracking process is used to obtain the final decomposition result. The backtracking scheme is similar to that used in sequence alignment.

Algorithm 3: **Expand function**

 **input**: Wavefront *W*, cost score *c*, gap/mismatch penalty score *g*/*h*

1 **Function** Expand(W, c):

2  **for** 1 ← *j* to *t* **do**

3    **for** 1 − *l*_*j*_ ← *d* to *n* − 1 **do**

4     $W_{c,j,d}=\max\{W_{c-h,j,d}+1,W_{c-g,j,d+1},W_{c-g,j,d-1}+1\}$

### Runtime complexity and memory footprint

We assume that the tandem repeat sequence has length *n*, and that *t* templates are provided, each of length at most *n*. Let *c* denote the total decomposition cost. Under these assumptions, the theoretical time complexity of WSD is *O*(*tnc*) and the space complexity is *O*(*tc*^2^).

In WSD, the *t* templates correspond to *t* DP matrices. For a single DP matrix, the expand procedure activates *O*(*c*) diagonals, each of which triggers an extend operation. Because each extend operation performs *O*(*n*) work, the computation per DP matrix is bounded by *O*(*nc*). As shown in Algorithm 1, WSD performs *O*(*c*) iterations. If the cost at iteration *i* is *s* (0 ≤ *s* ≤ *c*), then *O*(*s*) diagonals contain offsets that must be stored. Summed over all iterations, the total number of stored offsets is bounded by $\sum_{s=0}^{c}O(s)=O(c^{2})$. Considering all *t* DP matrices, the overall time and space complexities are therefore *O*(*tnc*) and *O*(*tc*^2^), respectively.

Empirical evaluation on simulated data sets supports this analysis: the running time of WSD scales approximately linearly with both *c* and *t*, while memory usage grows linearly with *t* (Fig. 2E and Supplemental Fig. S3). The theoretically expected quadratic dependence on *c* in memory usage was not prominent in practice. Similar behavior was also observed for the WFA algorithm (Marco-Sola et al. 2021).

### WSD algorithm: two adaptive strategies

We employ two strategies to accelerate the WSD algorithm. The first strategy leverages a simple yet highly effective adaptive approach to partition long tandem repeat sequences into shorter subsequences. The decomposition results of these subsequences are then integrated to obtain the final decomposition of the long tandem repeat sequences. This approach eliminates the need for user-defined hyperparameters while ensuring decomposition accuracy (see Results section). Specifically, we determine the subsequence length based on the input template length. Given a maximum template sequence length *L*, the subsequence length is set to 2*L*. Because sequence partitioning primarily affects the last block of each subsequence decomposition, the starting position of the next subsequence in the long tandem sequence is aligned with the starting point of the last block in the previous subsequence. Additionally, the WSD algorithm incorporates a parallelization scheme for ultra-long tandem repeat assemblies. These long sequences are divided into subsequences of length 1000*L* with an overlap of 10*L*. Each subsequence can be decomposed in parallel, and their results are then integrated to obtain the final decomposition.

The second strategy involves pruning the WSD algorithm itself. As the cost score increases, the wavefront set in WSD expands, leading to a growing number of diagonals that require calculation via the extend and expand functions. During iterations, the algorithm tracks the maximum offset. When the cost score reaches a predefined threshold (default: 10), if the difference between the current diagonal’s offset and the maximum offset exceeds a predefined limit (default: 100), the diagonal is deemed unlikely to be part of the optimal path. The default parameters were chosen empirically during tool development. Although not based on a comprehensive parameter search, large-scale simulations confirmed that these settings are robust across a wide range of sequence lengths, repeat architectures, and mutation rates. Further details on all parameters are provided in Supplemental Note 4.

To further quantify the contributions of the two adaptive strategies, we performed an ablation study. The results show that both strategies accelerate the WSD algorithm and maintain accurate decomposition results, with the first adaptive strategy providing the most substantial improvement (see Supplemental Tables S13–S15). Specifically, compared with the version without any adaptive strategies, using only the first adaptive resulted in an approximately 400-fold speedup while reducing memory usage to about 1/100 of the original. Using only the second adaptive strategy produced a more modest performance gain of about 1.5-fold and reduced memory usage to roughly one quarter of the original.

## Code availability

The source code of WSD is available at GitHub (https://github.com/junhaiqi/wsd) under the MIT License and as Supplemental Code. All benchmarking data and scripts required to reproduce this work are available at Figshare (https://doi.org/10.6084/m9.figshare.30818273) and as Supplemental Data.

## Competing interest statement

The authors declare no competing interests.

## Supplemental Material

Supplement 1

Supplement 2

## References

[GR281346QIC1] Bahk K, Sung J. 2024. SiAlign: an alignment algorithm guided by explicit similarity criteria. Nucleic Acids Res 52: 8717–8733. 10.1093/nar/gkae607 39011889 PMC11347165

[GR281346QIC2] Benson G. 1999. Tandem repeats finder: a program to analyze DNA sequences. Nucleic Acids Res 27: 573–580. 10.1093/nar/27.2.573 9862982 PMC148217

[GR281346QIC3] Bzikadze AV, Pevzner PA. 2020. Automated assembly of centromeres from ultra-long error-prone reads. Nat Biotechnol 38: 1309–1316. 10.1038/s41587-020-0582-4 32665660 PMC10718184

[GR281346QIC4] Depienne C, Mandel JL. 2021. 30 years of repeat expansion disorders: What have we learned and what are the remaining challenges? Am J Hum Genet 108: 764–785. 10.1016/j.ajhg.2021.03.011 33811808 PMC8205997

[GR281346QIC5] Dvorkina T, Bzikadze AV, Pevzner PA. 2020. The string decomposition problem and its applications to centromere analysis and assembly. Bioinformatics 36: i93–i101. 10.1093/bioinformatics/btaa454 32657390 PMC7428072

[GR281346QIC6] Gao S, Yang X, Guo H, Zhao X, Wang B, Ye K. 2023. HiCAT: A tool for automatic annotation of centromere structure. Genome Biol 24: 58. 10.1186/s13059-023-02900-5 36978122 PMC10053651

[GR281346QIC7] Gao S, Zhang Y, Bush SJ, Wang B, Yang X, Ye K. 2024. Centromere landscapes resolved from hundreds of human genomes. Genom Proteomics Bioinformatics 22: qzae071. 10.1093/gpbjnl/qzae071 PMC11652271 39423139

[GR281346QIC8] Gotoh O. 1982. An improved algorithm for matching biological sequences. J Mol Biol 162: 705–708. 10.1016/0022-2836(82)90398-9 7166760

[GR281346QIC9] Harris RS, Cechova M, Makova KD. 2019. Noise-cancelling repeat finder: uncovering tandem repeats in error-prone long-read sequencing data. Bioinformatics 35: 4809–4811. 10.1093/bioinformatics/btz484 31290946 PMC6853708

[GR281346QIC10] Ichikawa K, Kawahara R, Asano T, Morishita S. 2023. A landscape of complex tandem repeats within individual human genomes. Nat Commun 14: 5530. 10.1038/s41467-023-41262-1 37709751 PMC10502081

[GR281346QIC11] Kunyavskaya O, Dvorkina T, Bzikadze AV, Alexandrov IA, Pevzner PA. 2022. Automated annotation of human centromeres with hormon. Genome Res 32: 1137–1151. 10.1101/gr.276362.121 35545449 PMC9248890

[GR281346QIC12] Li H, Durbin R. 2024. Genome assembly in the telomere-to-telomere era. Nat Rev Genet 25: 658–670. 10.1038/s41576-024-00718-w 38649458

[GR281346QIC13] Logsdon GA, Rozanski AN, Ryabov F, Potapova T, Shepelev VA, Catacchio CR, Porubsky D, Mao Y, Yoo D, Rautiainen M, 2024. The variation and evolution of complete human centromeres. Nature 629: 136–145. 10.1038/s41586-024-07278-3 38570684 PMC11062924

[GR281346QIC14] Marco-Sola S, Moure JC, Moreto M, Espinosa A. 2021. Fast gap-affine pairwise alignment using the wavefront algorithm. Bioinformatics 37: 456–463. 10.1093/bioinformatics/btaa777 32915952 PMC8355039

[GR281346QIC15] Marco-Sola S, Eizenga JM, Guarracino A, Paten B, Garrison E, Moreto M. 2023. Optimal gap-affine alignment in *O*(*s*) space. Bioinformatics 39: btad074. 10.1093/bioinformatics/btad074 36749013 PMC9940620

[GR281346QIC16] Masutani B, Kawahara R, Morishita S. 2023. Decomposing mosaic tandem repeats accurately from long reads. Bioinformatics 39: btad185. 10.1093/bioinformatics/btad185 37039842 PMC10118999

[GR281346QIC17] Naish M, Alonge M, Wlodzimierz P, Tock AJ, Abramson BW, Schmücker A, Mandáková T, Jamge B, Lambing C, Kuo P, 2021. The genetic and epigenetic landscape of the Arabidopsis centromeres. Science 374: eabi7489. 10.1126/science.abi7489 34762468 PMC10164409

[GR281346QIC18] Needleman SB, Wunsch CD. 1970. A general method applicable to the search for similarities in the amino acid sequence of two proteins. J Mol Biol 48: 443–453. 10.1016/0022-2836(70)90057-4 5420325

[GR281346QIC19] Nurk S, Koren S, Rhie A, Rautiainen M, Bzikadze AV, Mikheenko A, Vollger MR, Altemose N, Uralsky L, Gershman A, 2022. The complete sequence of a human genome. Science 376: 44–53. 10.1126/science.abj6987 35357919 PMC9186530

[GR281346QIC20] Park J, Kaufman E, Valdmanis PN, Bafna V. 2023. TRviz: a Python library for decomposing and visualizing tandem repeat sequences. Bioinform Adv 3: vbad058. 10.1093/bioadv/vbad058 37168281 PMC10166586

[GR281346QIC21] Qi J, Ma J, Han R, Han Z, Yu T, Li G. 2025. De novo annotation of centromere with centroAnno. bioRxiv 10.1101/2025.02.19.639205

[GR281346QIC22] Ren J, Gu B, Chaisson MJ. 2023. vamos: variable-number tandem repeats annotation using efficient motif sets. Genome Biol 24: 175. 10.1186/s13059-023-03010-y 37501141 PMC10373352

[GR281346QIC23] Ting DT, Lipson D, Paul S, Brannigan BW, Akhavanfard S, Coffman EJ, Contino G, Deshpande V, Iafrate AJ, Letovsky S, 2011. Aberrant overexpression of satellite repeats in pancreatic and other epithelial cancers. Science 331: 593–596. 10.1126/science.1200801 21233348 PMC3701432

[GR281346QIC24] Zhang P, Wei Y, Tian Q, Zou Q, Wang Y. 2025. Fast sequence alignment for centromere with RaMA. Genome Res 35: 1209–1218. 10.1101/gr.279763.124 39939176 PMC12047532

