# Supplementary material for: Enabling efficient and robust analysis of tandem repeats in genomic data using Wavefront-based String Decomposer
Source: Genome Res. 2026 Jun;36(6):1265–72. doi: 10.1101/gr.281346.125 (PMC13262951; doi:10.1101/gr.281346.125)
Supplement: Supplement 1 [file Supplemental_Material.zip › SupplementalMaterial/Supplementary_Material.pdf]

Supplementary Tables

Supplemental Table S1. 36 different types of simulated assembly datasets. The details of the generation are in Supplementary Note 2.

Dataset	Monomer Template Length	Template Difference Rate	Mutation Rate
**S1**	100	0.1	0.005
**S2**	100	0.1	0.015
**S3**	100	0.1	0.025
**S4**	100	0.2	0.005
**S5**	100	0.2	0.015
**S6**	100	0.2	0.025
**S7**	100	0.3	0.005
**S8**	100	0.3	0.015
**S9**	100	0.3	0.025
**S10**	200	0.1	0.005
**S11**	200	0.1	0.015
**S12**	200	0.1	0.025
**S13**	200	0.2	0.005
**S14**	200	0.2	0.015
**S15**	200	0.2	0.025
**S16**	200	0.3	0.005
**S17**	200	0.3	0.015
**S18**	200	0.3	0.025
**S19**	300	0.1	0.005
**S20**	300	0.1	0.015
**S21**	300	0.1	0.025
**S22**	300	0.2	0.005
**S23**	300	0.2	0.015
**S24**	300	0.2	0.025
**S25**	300	0.3	0.005
**S26**	300	0.3	0.015
**S27**	300	0.3	0.025
**S28**	400	0.1	0.005
**S29**	400	0.1	0.015
**S30**	400	0.1	0.025
**S31**	400	0.2	0.005
**S32**	400	0.2	0.015
**S33**	400	0.2	0.025
**S34**	400	0.3	0.005
**S35**	400	0.3	0.015
**S36**	400	0.3	0.025

Supplemental Table S2. 24 different types of simulated sequencing datasets. The details of the generation are in Supplementary Note 2.

Dataset	Read Length	Sequencing Error Rate
**SR1**	5000	0.01
**SR2**	5000	0.03
**SR3**	5000	0.05
**SR4**	5000	0.1
**SR5**	5000	0.15
**SR6**	5000	0.2
**SR7**	10000	0.01
**SR8**	10000	0.03
**SR9**	10000	0.05
**SR10**	10000	0.1
**SR11**	10000	0.15
**SR12**	10000	0.2
**SR13**	15000	0.01
**SR14**	15000	0.03
**SR15**	15000	0.05
**SR16**	15000	0.1
**SR17**	15000	0.15
**SR18**	15000	0.2
**SR19**	20000	0.01
**SR20**	20000	0.03
**SR21**	20000	0.05
**SR22**	20000	0.1
**SR23**	20000	0.15
**SR24**	20000	0.2

Supplemental Table S3. Running time and memory usage of all decomposition methods based on monomers as templates on simulated assembly datasets.

Dataset	Monomer Decomposition					
	Elapsed Time (s)			Peak RAM (MB)		
	StringDecomposer	WSD	Speedup	StringDecomposer	WSD	Usage Ratio
**S1**	4.95	0.44	11.25	1637.38	40.71	40.22
**S2**	4.86	1.60	3.04	1637.35	43.99	37.22
**S3**	4.78	1.97	2.43	1637.32	41.30	39.64
**S4**	5.12	0.29	17.66	1637.37	40.77	40.16
**S5**	5.02	1.12	4.48	1637.34	46.12	35.50
**S6**	4.86	1.63	2.98	1637.29	41.18	39.76
**S7**	4.83	0.31	15.58	1637.33	40.68	40.25
**S8**	4.75	1.12	4.24	1637.36	50.42	32.48
**S9**	5.05	1.59	3.18	1637.35	41.29	39.65
**S10**	11.39	1.93	5.90	3152.00	57.10	55.20
**S11**	11.65	3.69	3.16	3152.02	51.05	61.74
**S12**	11.75	5.76	2.04	3152.05	52.02	60.59
**S13**	12.09	1.95	6.20	3151.99	50.30	62.66
**S14**	11.63	3.44	3.38	3152.04	50.96	61.85
**S15**	12.71	5.07	2.51	3152.01	51.97	60.65
**S16**	12.20	2.20	5.55	3151.97	64.77	48.66
**S17**	11.41	3.52	3.24	3151.88	51.18	61.58
**S18**	11.86	4.69	2.53	3151.88	52.07	60.53
**S19**	19.71	2.61	7.55	4666.68	71.50	65.27
**S20**	20.50	6.41	3.20	4666.76	65.73	71.00
**S21**	19.03	11.47	1.66	4666.57	68.34	68.29
**S22**	17.43	2.98	5.85	4666.72	86.50	53.95
**S23**	17.75	6.29	2.82	4666.66	65.71	71.02
**S24**	19.78	11.59	1.71	4666.58	66.80	69.85
**S25**	18.24	3.17	5.75	4666.74	95.34	48.95
**S26**	19.34	5.62	3.44	4666.75	65.75	70.97
**S27**	18.42	8.43	2.19	4666.75	66.80	69.86
**S28**	27.77	4.63	6.00	6181.18	68.65	90.04
**S29**	26.53	12.14	2.19	6181.24	70.39	87.81
**S30**	27.52	14.17	1.94	6181.11	74.29	83.21
**S31**	28.06	4.56	6.15	6181.25	68.65	90.04
**S32**	26.90	9.89	2.72	6181.29	70.27	87.97
**S33**	26.84	15.81	1.70	6181.25	74.30	83.19
**S34**	26.87	4.20	6.40	6181.22	68.57	90.15
**S35**	27.52	9.76	2.82	6181.26	70.25	87.98
**S36**	29.89	15.54	1.92	6181.18	74.73	82.71

Supplemental Table S4. Running time and memory usage of all decomposition methods based on a single HOR as templates on simulated assembly datasets.

Dataset	HOR Decomposition					
	Elapsed Time (s)			Peak RAM (MB)		
	StringDecomposer	WSD	Speedup	StringDecomposer	WSD	Usage Ratio
**S1**	4.30	0.08	53.75	1557.33	18.81	82.80
**S2**	4.56	0.83	5.49	1557.38	19.83	78.53
**S3**	4.45	1.20	3.71	1557.38	19.49	79.90
**S4**	4.69	0.07	67.00	1557.31	18.82	82.73
**S5**	4.31	0.60	7.18	1557.40	18.95	82.19
**S6**	4.36	1.17	3.73	1557.38	19.55	79.64
**S7**	4.26	0.07	60.86	1557.25	18.86	82.55
**S8**	4.46	0.60	7.43	1557.30	19.81	78.60
**S9**	4.46	1.15	3.88	1557.32	19.46	80.01
**S10**	11.03	1.24	8.90	3072.45	97.26	31.59
**S11**	10.61	3.55	2.99	3072.24	90.64	33.89
**S12**	11.31	5.20	2.17	3072.21	90.62	33.90
**S13**	10.90	1.34	8.13	3072.32	99.01	31.03
**S14**	10.57	3.32	3.18	3072.31	93.70	32.79
**S15**	11.21	4.95	2.26	3072.29	90.79	33.84
**S16**	10.46	1.59	6.58	3072.27	94.77	32.42
**S17**	10.72	3.92	2.73	3072.31	93.90	32.72
**S18**	10.49	5.27	1.99	3072.20	92.28	33.29
**S19**	16.55	1.86	8.90	4586.76	101.75	45.08
**S20**	16.25	6.77	2.40	4586.66	117.61	39.00
**S21**	17.16	10.04	1.71	4586.73	105.57	43.45
**S22**	17.12	2.01	8.52	4586.87	103.07	44.50
**S23**	16.85	6.24	2.70	4586.71	116.70	39.30
**S24**	16.42	11.70	1.40	4586.77	103.18	44.45
**S25**	16.87	1.68	10.04	4586.82	103.38	44.37
**S26**	17.68	6.50	2.72	4586.84	111.75	41.05
**S27**	17.25	9.98	1.73	4586.80	103.07	44.50
**S28**	24.70	4.81	5.14	6101.44	108.00	56.49
**S29**	22.48	13.01	1.73	6101.49	125.55	48.60
**S30**	24.79	14.72	1.68	6101.48	113.00	54.00
**S31**	25.03	4.37	5.73	6101.45	115.43	52.86
**S32**	23.68	10.08	2.35	6101.59	121.92	50.05
**S33**	25.31	15.34	1.65	6101.50	121.60	50.18
**S34**	24.74	4.31	5.74	6101.48	112.37	54.30
**S35**	24.42	9.96	2.45	6101.50	107.12	56.96
**S36**	24.63	14.79	1.67	6101.50	115.15	52.99

Supplemental Table S5. Accuracy and bias of all decomposition methods based on monomers as templates on simulated assembly datasets.

Dataset	Accuracy		Bias	
	StringDecomposer	WSD	StringDecomposer	WSD
S1	1.00	1.00	0.00	0.00
S2	1.00	1.00	0.00	0.00
S3	1.00	1.00	0.00	0.00
S4	1.00	1.00	0.00	0.00
S5	1.00	1.00	0.00	0.00
S6	1.00	1.00	0.00	0.00
S7	1.00	1.00	0.00	0.00
S8	1.00	1.00	0.00	0.00
S9	1.00	1.00	0.00	0.00
S10	1.00	1.00	0.00	0.00
S11	1.00	1.00	0.00	0.00
S12	1.00	1.00	0.00	0.00
S13	1.00	1.00	0.00	0.00
S14	1.00	1.00	0.00	0.00
S15	1.00	1.00	0.00	0.00
S16	1.00	1.00	0.00	0.00
S17	1.00	1.00	0.00	0.00
S18	1.00	1.00	0.00	0.00
S19	1.00	1.00	0.00	0.00
S20	1.00	1.00	0.00	0.00
S21	1.00	1.00	0.00	0.00
S22	1.00	1.00	0.00	0.00
S23	1.00	1.00	0.00	0.00
S24	1.00	1.00	0.00	0.00
S25	1.00	1.00	0.00	0.00
S26	1.00	1.00	0.00	0.00
S27	1.00	1.00	0.00	0.00
S28	1.00	1.00	0.00	0.00
S29	1.00	1.00	0.00	0.00
S30	1.00	1.00	0.00	0.00
S31	1.00	1.00	0.00	0.00
S32	1.00	1.00	0.00	0.00
S33	1.00	1.00	0.00	0.00
S34	1.00	1.00	0.00	0.00
S35	1.00	1.00	0.00	0.00
S36	1.00	1.00	0.00	0.00

Supplemental Table S6. Accuracy and bias of all decomposition methods based on a single HOR as templates on simulated assembly datasets.

Dataset	Accuracy		Bias	
	StringDecomposer	WSD	StringDecomposer	WSD
S1	1.00	1.00	0.00	0.00
S2	1.00	1.00	0.00	0.00
S3	1.00	1.00	0.00	0.00
S4	1.00	1.00	0.00	0.00
S5	1.00	1.00	0.00	0.00
S6	1.00	1.00	0.00	0.00
S7	1.00	1.00	0.00	0.00
S8	1.00	1.00	0.00	0.00
S9	1.00	1.00	0.00	0.00
S10	0.00	1.00	700.00	0.00
S11	0.00	1.00	700.00	0.00
S12	0.00	1.00	700.00	0.00
S13	0.00	1.00	700.00	0.00
S14	0.00	1.00	700.00	0.00
S15	0.00	1.00	700.00	0.00
S16	0.00	1.00	700.00	0.00
S17	0.00	1.00	700.00	0.00
S18	0.00	1.00	700.00	0.00
S19	0.00	1.00	460.00	0.00
S20	0.00	1.00	402.00	0.00
S21	0.00	1.00	400.00	0.00
S22	0.00	1.00	462.00	0.00
S23	0.00	1.00	400.00	0.00
S24	0.00	1.00	400.00	0.00
S25	0.00	1.00	444.00	0.00
S26	0.00	1.00	401.00	0.00
S27	0.00	1.00	400.00	0.00
S28	0.00	1.00	800.00	0.00
S29	0.00	1.00	800.00	0.00
S30	0.00	1.00	800.00	0.00
S31	0.00	1.00	800.00	0.00
S32	0.00	1.00	800.00	0.00
S33	0.00	1.00	800.00	0.00
S34	0.00	1.00	800.00	0.00
S35	0.00	1.00	800.00	0.00
S36	0.00	1.00	800.00	0.00

Supplemental Table S7. Accuracy and bias of all decomposition methods based on monomers as templates on simulated sequencing datasets.

Dataset	Accuracy		Bias	
	StringDecomposer	WSD	StringDecomposer	WSD
SR1	1.00	1.00	0.00	0.00
SR2	1.00	1.00	0.00	0.00
SR3	1.00	1.00	0.00	0.00
SR4	1.00	1.00	0.00	0.00
SR5	1.00	1.00	0.00	0.00
SR6	1.00	1.00	0.00	0.00
SR7	1.00	1.00	0.00	0.00
SR8	1.00	1.00	0.00	0.00
SR9	1.00	1.00	0.00	0.00
SR10	1.00	1.00	0.00	0.00
SR11	1.00	1.00	0.00	0.00
SR12	1.00	1.00	0.00	0.00
SR13	1.00	1.00	0.00	0.00
SR14	1.00	1.00	0.00	0.00
SR15	1.00	1.00	0.00	0.00
SR16	1.00	1.00	0.00	0.00
SR17	1.00	1.00	0.00	0.00
SR18	1.00	1.00	0.00	0.00
SR19	1.00	1.00	0.00	0.00
SR20	1.00	1.00	0.00	0.00
SR21	1.00	1.00	0.00	0.00
SR22	1.00	1.00	0.00	0.00
SR23	1.00	1.00	0.00	0.00
SR24	1.00	1.00	0.00	0.00

Supplemental Table S8. Accuracy and bias of all decomposition methods based on a single HOR as templates on simulated sequencing datasets.

Dataset	Accuracy		Bias	
	StringDecomposer	WSD	StringDecomposer	WSD
SR1	1.00	1.00	0.00	0.00
SR2	1.00	1.00	0.00	0.00
SR3	1.00	1.00	0.00	0.00
SR4	1.00	1.00	0.00	0.00
SR5	1.00	1.00	0.00	0.00
SR6	1.00	1.00	0.00	0.00
SR7	1.00	1.00	0.00	0.00
SR8	1.00	1.00	0.00	0.00
SR9	1.00	1.00	0.00	0.00
SR10	1.00	1.00	0.00	0.00

**SR11**	1.00	1.00	0.00	0.00
**SR12**	1.00	1.00	0.00	0.00
**SR13**	1.00	1.00	0.00	0.00
**SR14**	1.00	1.00	0.00	0.00
**SR15**	1.00	1.00	0.00	0.00
**SR16**	1.00	1.00	0.00	0.00
**SR17**	1.00	1.00	0.00	0.00
**SR18**	1.00	1.00	0.00	0.00
**SR19**	1.00	1.00	0.00	0.00
**SR20**	1.00	1.00	0.00	0.00
**SR21**	1.00	1.00	0.00	0.00
**SR22**	1.00	1.00	0.00	0.00
**SR23**	1.00	1.00	0.00	0.00
**SR24**	1.00	1.00	0.00	0.00

Supplemental Table S9. Running time and memory usage of all decomposition methods based on monomers as templates on simulated sequencing datasets.

**Dataset**	**Monomer Decomposition**					
	Elapsed Time (s)			Peak RAM (MB)		
	StringDecomposer	WSD	Speedup	StringDecomposer	WSD	Usage Ratio
**SR1**	41.97	1.38	30.41	2865.64	48.09	59.59
**SR2**	15.01	2.75	5.46	2865.82	51.65	55.48
**SR3**	41.27	3.47	11.89	2865.91	53.73	53.34
**SR4**	43.40	6.54	6.64	2866.73	61.17	46.86
**SR5**	32.10	9.12	3.52	2867.46	73.12	39.22
**SR6**	27.08	15.92	1.70	2870.26	93.39	30.73
**SR7**	34.12	2.61	13.07	3154.22	54.73	57.63
**SR8**	36.58	4.62	7.92	3154.20	57.53	54.83
**SR9**	54.62	5.76	9.48	3154.22	59.66	52.87
**SR10**	47.76	11.66	4.10	3154.24	65.66	48.04
**SR11**	30.22	18.31	1.65	3154.16	79.07	39.89
**SR12**	42.22	29.39	1.44	3154.25	99.57	31.68
**SR13**	37.94	2.97	12.77	3131.88	60.75	51.55
**SR14**	44.76	6.76	6.62	3132.36	62.93	49.78
**SR15**	38.77	18.35	2.11	3148.33	64.93	48.49
**SR16**	37.26	16.30	2.29	3140.57	71.32	44.04
**SR17**	39.59	25.46	1.55	3139.11	84.12	37.32
**SR18**	38.30	44.67	0.86	3142.16	104.02	30.21
**SR19**	38.98	3.57	10.92	3166.27	67.25	47.08
**SR20**	49.18	7.64	6.44	3166.37	68.52	46.21
**SR21**	51.82	12.30	4.21	3166.31	69.84	45.34
**SR22**	46.89	20.94	2.24	3166.28	76.08	41.62

SR23	58.87	32.49	1.81	3166.11	90.16	35.12
**SR24**	52.74	49.27	1.07	3166.39	109.72	28.86

Supplemental Table S10. Running time and memory usage of all decomposition methods based on a single HOR as templates on simulated sequencing datasets.

**Dataset**	**HOR Decomposition**					
	Elapsed Time (s)			Peak RAM (MB)		
	StringDecomposer	WSD	Speedup	StringDecomposer	WSD	Usage Ratio
**SR1**	18.20	4.78	3.81	2794.05	112.24	24.89
**SR2**	13.90	9.63	1.44	2794.09	111.91	24.97
**SR3**	33.66	9.44	3.57	2795.18	111.59	25.05
**SR4**	10.26	8.71	1.18	2795.28	109.64	25.50
**SR5**	35.78	8.92	4.01	2795.79	111.49	25.08
**SR6**	13.34	10.66	1.25	2796.68	117.77	23.75
**SR7**	38.81	8.49	4.57	3909.03	116.65	33.51
**SR8**	44.01	16.31	2.70	3909.06	121.52	32.17
**SR9**	38.30	18.35	2.09	3909.06	117.26	33.34
**SR10**	20.75	18.28	1.14	3909.13	116.89	33.44
**SR11**	46.47	16.91	2.75	3909.23	118.97	32.86
**SR12**	50.32	21.43	2.35	3909.12	125.25	31.21
**SR13**	35.77	11.46	3.12	3852.00	126.75	30.39
**SR14**	52.11	24.31	2.14	3880.95	129.01	30.08
**SR15**	57.06	25.70	2.22	3881.91	123.46	31.44
**SR16**	54.80	24.67	2.22	3818.73	121.83	31.34
**SR17**	56.56	26.31	2.15	3913.69	125.43	31.20
**SR18**	81.40	31.50	2.58	3912.98	132.34	29.57
**SR19**	75.79	14.06	5.39	3921.03	130.51	30.04
**SR20**	66.38	31.46	2.11	3921.17	133.68	29.33
**SR21**	76.61	35.44	2.16	3921.06	129.14	30.36
**SR22**	32.34	38.16	0.85	3921.06	129.16	30.36
**SR23**	32.29	32.93	0.98	3921.13	130.40	30.07
**SR24**	28.67	37.37	0.77	3921.03	137.40	28.54

Supplemental Table S11. Block number and average identity of all decomposition methods on human centromeric assemblies. Decomposition Distance is calculated by Edlib (Šošić & Šikić, 2017).

**Chromosome**	**Block Number**		**Decomposition Distance**	
	StringDecomposer	WSD	StringDecomposer	WSD
**1**	26519	26504	54817	54645
**2**	13774	13772	46592	46592
**3**	5044	5044	6523	6553
**4**	4283	4283	6893	6898
**5**	15055	15055	33396	33391

**6**	16315	16315	10308	10308
**7**	19384	19378	21223	21167
**8**	12415	12412	18477	18645
**9**	15464	15462	38428	38384
**10**	11967	11967	25386	25431
**11**	19718	19718	44789	44789
**12**	15206	15206	31776	31732
**13**	11479	11478	9969	9924
**14**	15349	15348	25415	25585
**15**	5963	5963	9332	9340
**16**	11422	11421	21588	21585
**17**	21185	21184	28393	28658
**18**	28110	28110	54761	54761
**19**	23048	23046	65523	65502
**20**	12793	12793	19887	19887
**21**	2007	2006	4808	4765
**22**	17146	17146	28555	28567
**X**	18135	18134	33046	33110

Supplemental Table S12. Block number and average identity of all decomposition methods on Arabidopsis centromeric assemblies. Decomposition Distance is calculated by Edlib.

**Chromosome**	**Block Number**		**Decomposition Distance**	
	StringDecomposer	WSD	StringDecomposer	WSD
**1**	26519	26504	51070	51244
**2**	13774	13772	42860	42805
**3**	5044	5044	40643	40537
**4**	4283	4283	53363	53257
**5**	15055	15055	22234	22380

Supplemental Table S13. Ablation studies using two adaptive strategies in WSD: impact of adaptive strategy on accuracy. The experiment was conducted on S2.

**Adaptive Strategy**	**Accuracy**	**Bias**
**None**	1.00	0.00
**1**	1.00	0.00
**2**	1.00	0.00
**1 + 2**	1.00	0.00

Supplemental Table S14. Ablation studies using two adaptive strategies in WSD: impact of adaptive strategy on runtime and peak memory. The experiment was conducted on S2.

**Adaptive Strategy**	**Elapsed Time (s)**	**Peak RAM (MB)**
**None**	437.02	4620.52
**1**	1.06	44.96
**2**	280.49	1064.5

**1 + 2**	1.03	43.7

Supplemental Table S15. Ablation studies using two adaptive strategies in WSD: impact of adaptive strategy on complexity.

**Dataset**	**Monomer Decomposition**			
	**Elapsed Time (s)**		**Peak RAM (MB)**	
Two adaptive strategies	**Use**	**Do not use**	**Use**	**Do not use**
SR1	1.38	29.51	48.09	610.53
SR2	2.75	73.52	51.65	1900.72
SR3	3.47	107.71	53.73	2470.18
SR4	6.54	105.43	61.17	3039.85
SR5	9.12	182.6	73.12	3361.58
SR6	15.92	154.87	93.39	3542.67

Supplementary Figures

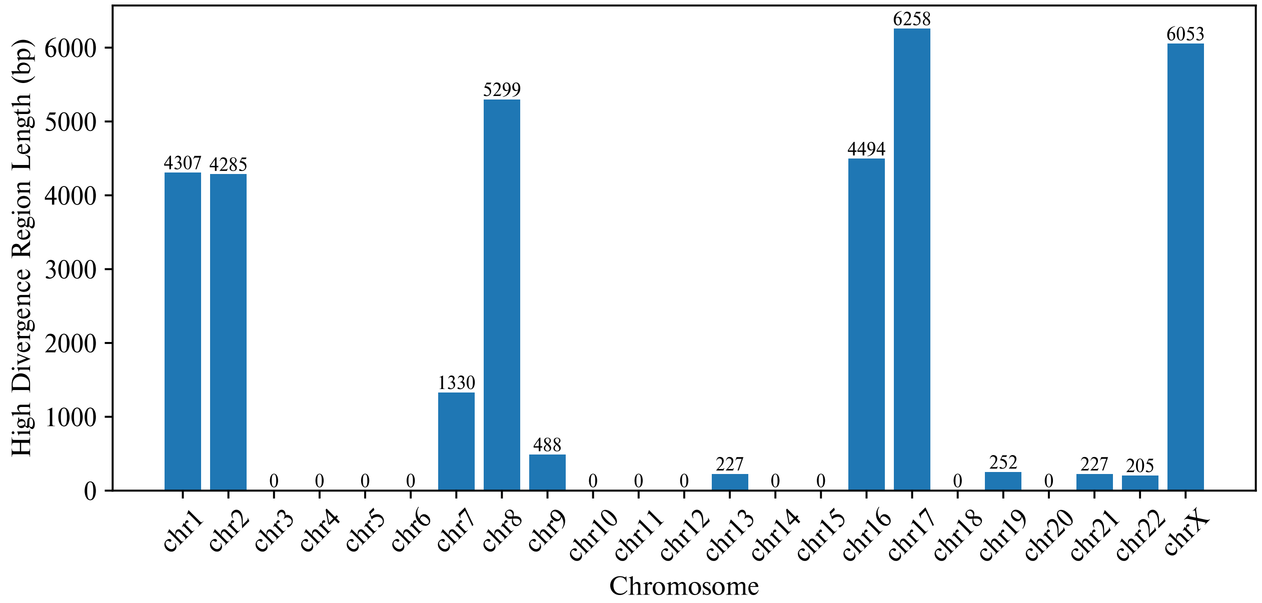

Supplemental Figure S1. Length distribution of highly divergent regions detected by the WSD algorithm on the human CHM13 (v2.0) genome. Each block in the WSD output has an identity score; a lower identity score indicates higher divergence from the corresponding template. All blocks with an identity score less than 0.7 were defined as high-divergence regions.

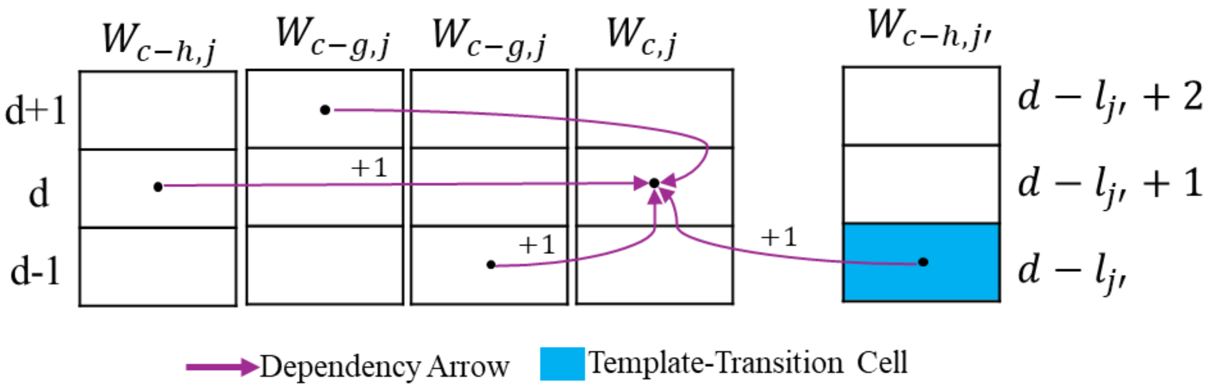

Supplemental Figure S2. Dependencies between wavefronts as to compute $W_{c,j,d}$ (Equation 8, 10). Dependencies introduced by the template-transition cell in the rightmost column are only allowed when the DP-cell index k=1, which corresponds to the first position of a template, as defined in the last line of Equation 8. Taking this edge implies that the extension cannot be handled by an LCP-based continuation. If the character at the current position of the tandem repeat sequence were compatible with the first character of the new template, the extension would have been absorbed by the LCP mechanism. Therefore, using the template-transition edge necessarily indicates a mismatch, which justifies the penalty h. For this reason, the rightmost column is defined as $W_{c-h,j'}$, as shown in the last line of Equation 10.

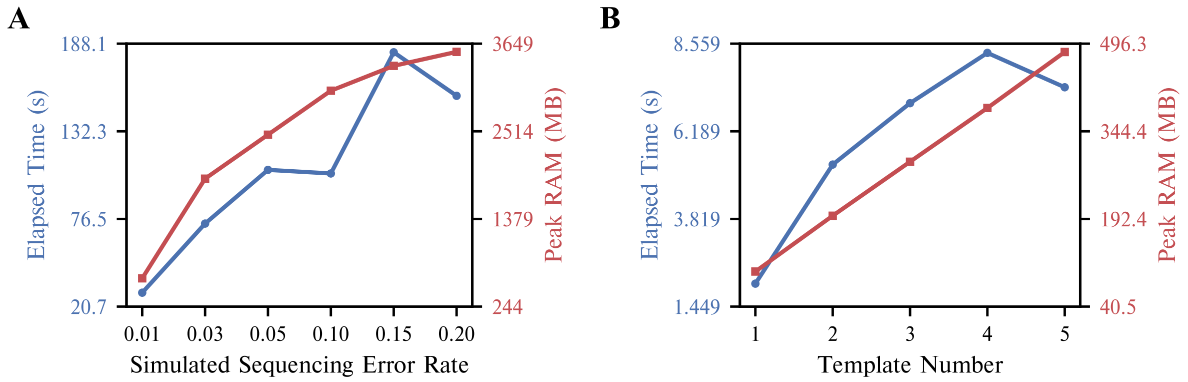

Supplemental Figure S3. WSD's runtime and peak memory under different simulation sequencing error rates and different template numbers. WSD did not use either of the two adaptive strategies; the test datasets were SR1-SR6 (Supplemental Figure S3A, running WSD using monomers as templates) and SR1 (Supplemental Figure S3B, running WSD using a single HOR as a template).

Supplementary Notes

1. **Experimental environment and command lines**
2. **Generation of simulated datasets**
3. **An equivalence proof for Algorithm 3 and Equation 10**
4. **Selection of WSD parameters**

Supplementary Note 1: Experimental environment and command lines

All experiments were conducted on a CentOS 7.9.2 system equipped with an Intel (R) Xeon(R) Platinum 8358P CPU and 512 GB of memory. The experiments did not involve the use of a GPU.

The command lines on simulated datasets:

StringDecomposer (v1.1.2) on S1-S36: `stringdecomposer $ref.fa $templates.fa -t 36`

WSD (v.1.0.1) on S1-S36, SR1-SR24: `./wsd $ref.fa -m $templates.fa -t 36`

StringDecomposer (v1.1.2) with Parameter 1 on S1-S36:
`stringdecomposer $ref.fa $templates.fa -t 36 -b 7500 -v 1000`

StringDecomposer (v1.1.2) with Parameter 2 on S1-S36:
`stringdecomposer $ref.fa $templates.fa -t 36 -b 10000 -v 2000`

StringDecomposer (v1.1.2) on SR1-SR24:
`stringdecomposer $ref.fa $templates.fa -t 36 -v 2000`

The command lines on Human/Arabidopsis centromeric assemblies:

StringDecomposer (v1.1.2): `stringdecomposer $ref.fa $templates.fa -t 36`

WSD (v.1.0.1): `./wsd $ref.fa -m $templates.fa -t 36 -A1`

The command lines of WSD on SR1-SR6 (closing two adaptive strategies):
`./wsd $ref.fa -m $templates.fa -t 36 -c 1000000 -d 1000000 -a 0 -b 1000000`

Supplementary Note 2: Generation of simulated datasets

S1-S36. We generated simulated assembly datasets directly using the HiCAT source code. First, five random sequences of lengths 100, 200, 300, and 400 bp were generated to serve as raw monomer templates (Monomer Template Length). These five monomers were concatenated to form a single higher-order repeat (HOR). Each monomer within the HOR was then independently mutated at rates of 10%, 20%, or 30% (Template Difference Rate). The resulting five mutated monomers were concatenated to reconstruct the HOR, which was subsequently repeated 40 times to generate a full assembly. During this repetition, each HOR unit was further mutated at rates of 0.5%, 1.5%, or 2.5% (Mutation Rate). This entire simulation process was repeated 100 times, producing 100 assemblies for each of the S1-S36 datasets.

SR1-SR24. For read-level simulations, we generated fixed-length reads from the single HOR sequence (1000 bp) corresponding to S18. This HOR sequence was repeated 5, 10, 15, or 20 times to produce sequences of 5,000, 10,000, 15,000, and 20,000 bp, respectively. To simulate varying sequencing error

rates, mutations were introduced at rates of 0.01, 0.03, 0.05, 0.1, 0.15, and 0.2. The simulation was repeated 500 times for each configuration, resulting in 500 simulated reads for each of the SR1-SR24 datasets.

Supplementary Note 3: An equivalence proof for Algorithm 3 and Equation 10

There is a subtle but important detail here. Algorithm 3 suggests that we first determine the maximum among $W_{c-h,j,d}+1, W_{c-g,j,d+1}$ and $W_{c-g,j,d-1}+1$, and then perform the extend procedure starting from this maximum value. In contrast, Equation 10 implies that the extend procedure should be executed starting from all three values $W_{c-h,j,d}+1, W_{c-g,j,d+1}$, and $W_{c-g,j,d-1}+1$, followed by taking the maximum of their extension results. We claim that these two approaches are equivalent; that is,

$$ETP(\max(a,b),R,M) = \max(ETP(a,R,M),ETP(b,R,M))$$

To prove this, assume without loss of generality that $b \geq a$. Then it suffices to show that

$$\max(ETP(a,R,M),ETP(b,R,M)) = ETP(b,R,M)$$

When performing extend from position a, there are two possible outcomes:

1. **The extension from a can reach b, meaning no mismatch occurs between a and b.**
   In this case, the extension continues identically from b, so

$$ETP(a,R,M) = ETP(b,R,M) = \max(ETP(a,R,M),ETP(b,R,M))$$

2. **The extension from a cannot reach b, i.e., a mismatch occurs before reaching b.**
   Then the extension must stop earlier, and therefore

$$\max(ETP(a,R,M),ETP(b,R,M)) = ETP(b,R,M)$$

In both cases, we obtain

$$\max(ETP(a,R,M),ETP(b,R,M)) = ETP(b,R,M)$$

Thus,

$$ETP(\max(a,b),R,M) = \max(ETP(a,R,M),ETP(b,R,M))$$

and therefore the two formulations are equivalent.

Supplementary Note 4: Selection of WSD parameters

WSD provides several user-configurable parameters that control its performance, accuracy, and memory usage. Table S1 summarizes these parameters, followed by a discussion of how each

parameter affects algorithmic behavior and how users may select appropriate values depending on their application.

Table S1. The parameters of WSD algorithm.

**-m**	Specify the template file path with fasta type [required parameter]
**-c**	Specify the cost score threshold for fast wave extension [default = 10]
**-d**	Specify the max offset distance threshold for fast wave extension [default = 100]
**-b**	Specify the batch size for fast wave extension [default = 1000]
**-a**	Specify whether (1: yes, 0: no) to adaptively determine the batch size [default = 1]
**-t**	Specify the thread number [default = 1]
**-A**	Specify whether (1: yes, 0: no) to decompose ultralong tandem repeat assemblies [default = 0]
**-M**	Specify the mismatch penalty score [default = 1]
**-G**	Specify the gap/insertion penalty score [default = 1]

1. **-c:** When the decomposition cost reaches this parameter value, the second adaptive strategy is activated. A lower threshold triggers this adaptive strategy earlier. For sequences that are highly similar to the templates, using a smaller value still preserves optimal decomposition. The default value (10) works well for centromeric TRs with divergence ≤ 10-20% and can be increased for more divergent sequences. It is worth noting that this parameter interacts with the mismatch (**-M**) and gap (**-G**) penalties: the ratio of the value to these penalty values approximately indicates the iteration at which the second adaptive strategy will be activated.
2. **-d:** When the difference between the offset on a diagonal and the maximum offset (the maximum value of the offsets on all diagonals) exceeds this value, this diagonal is discarded and excluded from subsequent extend and expand operations. Smaller values restrict the search space and improve speed, but may cause failures when long insertions or deletions are present. Larger values increase robustness to long indels but at the cost of higher runtime and memory usage. The default setting works well for human α-satellite sequences; increasing this value is recommended only when long insertions are expected.
3. **-b:** To accelerate string decomposition, long sequences are often partitioned into shorter subsequences, which are decomposed independently and later merged into the final result. This parameter value specifies the length of each subsequence. Note that it takes effect only when the adaptive partitioning strategy is disabled (i.e., when **-a 0**). This parameter should depend on the maximum length of the template monomers. To maintain accuracy, the subsequence length must be strictly greater than the maximum template length, and a safer choice is to set it to at least 1.5× the maximum template length.
4. **-a:** If the value is set to 1, the subsequence length is determined adaptively (set to twice the maximum template length). We recommend keeping this value at the default value of 1.
5. **-A:** When decomposing ultra-long sequences (e.g., centromere assemblies) or sequences ≥ 100 kb, we recommend enabling this option by setting **-A 1**.
6. **-M, -G:** These parameters define the alignment scoring scheme. The default equal mismatch/gap penalty works well for most TR sequences; adjust them only when optimizing for specific biological features (e.g., long homopolymer indels in ONT reads).

References

Šošić, M., & Šikić, M. (2017). Edlib: a C/C++ library for fast, exact sequence alignment using edit distance. *Bioinformatics*, *33*(9), 1394-1395.
