# Supplementary material for: Enabling efficient and robust analysis of tandem repeats in genomic data using Wavefront-based String Decomposer
Source: Genome Res. 2026 Jun;36(6):1265–72. doi: 10.1101/gr.281346.125 (PMC13262951; doi:10.1101/gr.281346.125)
Supplement: Supplement 1 [file Supplemental_Material.zip › SupplementalMaterial/MainFigures/Main-Figure-1.pdf]

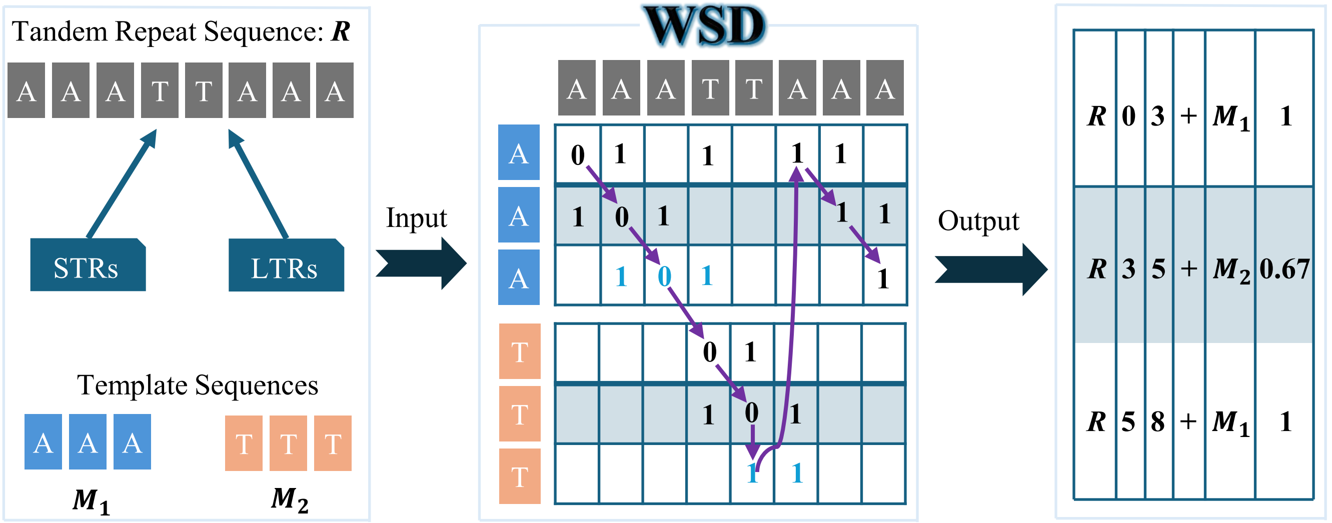

Tandem Repeat Sequence: R
A A A T T A A A
STRs
LTRs
Template Sequences
A A A
T T T
M1
M2
Input
WSD
Output
R 0 3 + M1 1
R 3 5 + M2 0.67
R 5 8 + M1 1
