# Supplementary figures and images for: Enabling efficient and robust analysis of tandem repeats in genomic data using Wavefront-based String Decomposer
Source: Genome Res. 2026 Jun;36(6):1265–72. doi: 10.1101/gr.281346.125 (PMC13262951; doi:10.1101/gr.281346.125)

**A****Monomer-based decomposition**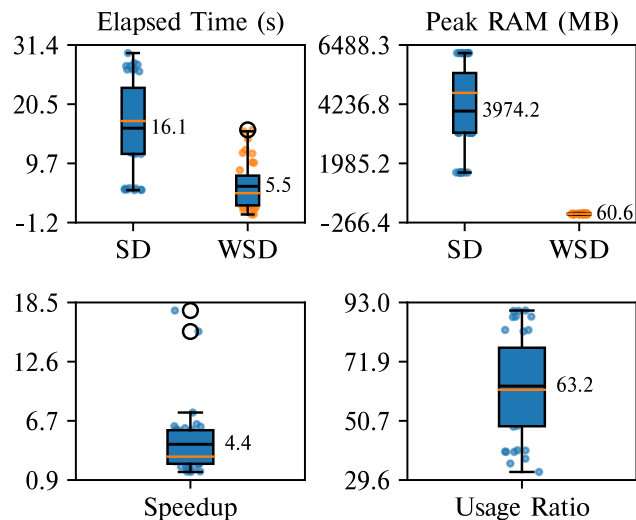**B****HOR-based decomposition**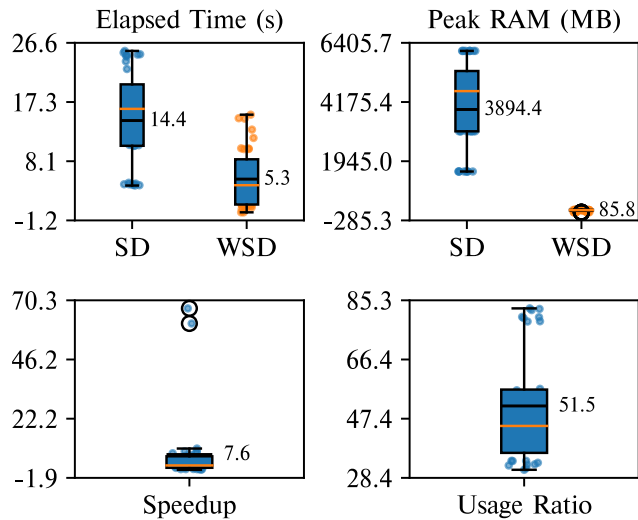**C**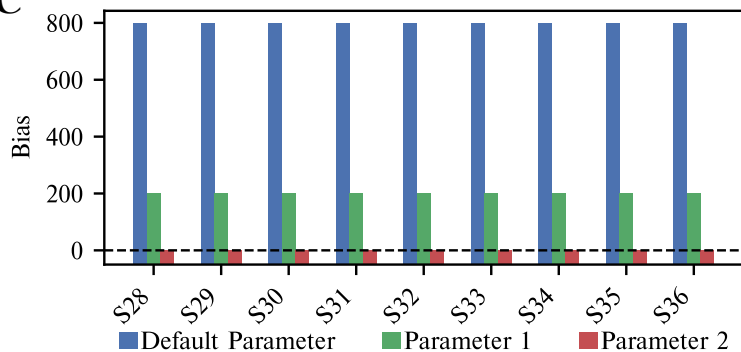**D**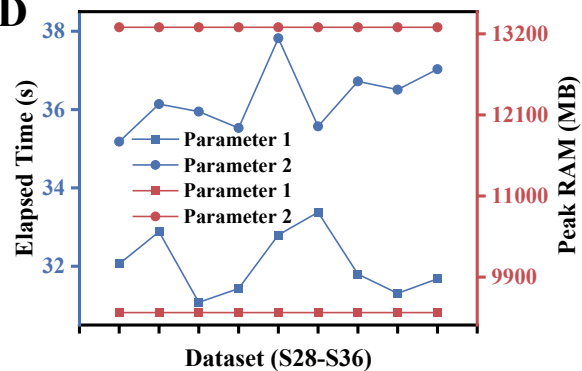**E**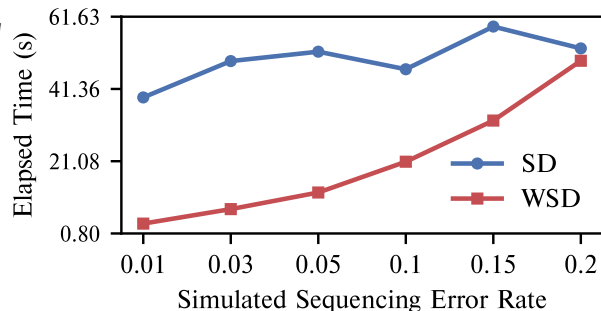**F**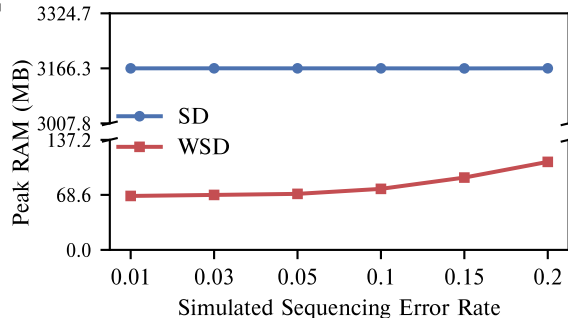

Supplement: Supplement 1 [file Supplemental_Material.zip › SupplementalMaterial/MainFigures/Main-Figure-2.pdf]

**A**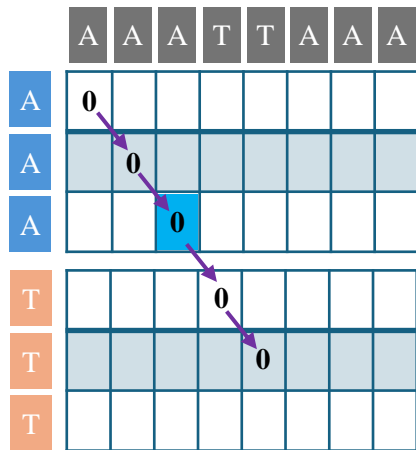**B**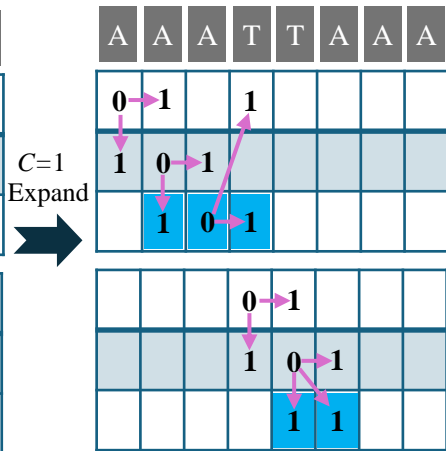**C**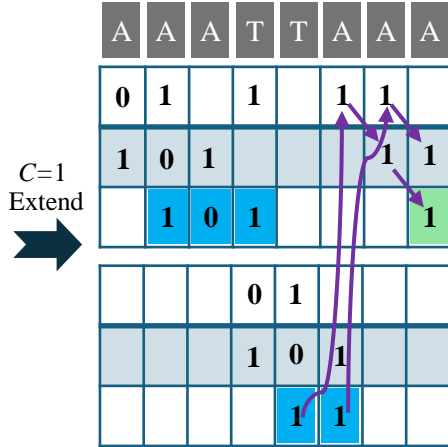

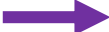 Extend Arrow
 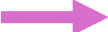 Expand Arrow
 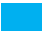 Template-Transition Cell
 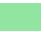 Final Cell

Supplement: Supplement 1 [file Supplemental_Material.zip › SupplementalMaterial/MainFigures/Main-Figure-3.pdf]
