# Supplementary material for: Enabling efficient and robust analysis of tandem repeats in genomic data using Wavefront-based String Decomposer
Source: Genome Res. 2026 Jun;36(6):1265–72. doi: 10.1101/gr.281346.125 (PMC13262951; doi:10.1101/gr.281346.125)
Supplement: Supplement 1 [file Response.pdf › SupplementalMaterial/Response.pdf]

==

Response to reviewers:

We are grateful to the reviewers for their thoughtful and thorough comments, which helped us improve our paper greatly. We have revised the paper following their comments. Below please find the point-by-point response to all the reviewers' comments, where "Q:" indicates the original comments (in blue color) and "Ans:" indicates our answers (in black color). The change of text in manuscript and Supplement is marked by red color.

Reviewer 2

Q: Comments to the Author

The authors have addressed my concerns. However, when aligning the sequences created by the aligner annotation there were large gaps in the reference, as shown by my attached alignment (see Supplemental Material). This was formed by concatenating the annotated sequences for Chr 10 (arbitrarily), and then aligning 10kb subsequences to CHM13.

Ans: We sincerely thank the reviewer for the careful evaluation and for raising this important point.

It is important to note that the completeness of sequence decomposition depends on the completeness of the input monomer library. This is a general property of methods that rely on predefined monomer sets, including WSD and StringDecomposer. When real centromeres contain highly divergent monomers or novel variants that are not represented in the input library, sequence similarity decreases and these regions may not be included in the reconstructed sequence. As a result, aligning the reconstructed sequence back to the genome can reveal large gaps, often with lengths that are multiples of the monomer size, corresponding to these unrepresented variants.

Consistent with this explanation, we observed that many of the large deletions in the reviewer's alignment correspond closely to integer multiples of the ~171 bp alpha-satellite monomer length (e.g., ~339 bp = 2 monomers; ~1019 bp = 6 monomers). To further investigate this pattern, we systematically analyzed all large deletions (> 50 bp) across the alignment records (see Table 1). The results show that the vast majority of these deletions match integer multiples of the monomer length.

These observations suggest that the gaps are not random alignment artifacts, but instead reflect missing or highly divergent monomer variants that are not represented in the input library, leading to their absence in the reconstructed sequence. Therefore, the observed gaps are a natural consequence of current monomer library limitations rather than inaccuracies of the WSD algorithm itself.

We agree that improving and expanding the monomer library to better capture centromere sequence diversity will be an important direction for reducing such gaps in future analyses, and we have now clarified this point in the revised **Discussion**.

Table 1. Statistical analysis of deletion values in the alignment results.

Unique Deletion Value (bp)	Monomers Count	Approximately Copies
**2717**	15.89	16
**2036**	11.91	12
**1698**	9.93	10
**1697**	9.92	10

**1696**	9.92	10
**1694**	9.91	10
**1359**	7.95	8
**1358**	7.94	8
**1357**	7.94	8
**1355**	7.92	8
**1351**	7.9	8
**1020**	5.96	6
**1019**	5.96	6
**1018**	5.95	6
**1017**	5.95	6
**1016**	5.94	6
**1015**	5.94	6
**683**	3.99	4
**680**	3.98	4
**679**	3.97	4
**678**	3.96	4
**677**	3.96	4
**675**	3.95	4
**509**	2.98	3
**508**	2.97	3
**507**	2.96	3
**341**	1.99	2
**340**	1.99	2
**339**	1.98	2
**338**	1.98	2
**337**	1.97	2
**336**	1.96	2
**335**	1.96	2
**171**	1	1
**170**	0.99	1
**169**	0.99	1
**79**	0.46	0

We have added the following paragraph to the **Discussion** to point out this issue:

“It is important to note that the completeness of sequence decomposition depends on the completeness of the input monomer library. This is a general property of methods that rely on predefined monomer sets, including WSD and StringDecomposer. When real centromeres contain highly divergent monomers or novel variants that are not represented in the input library, sequence similarity decreases and these regions may not be included in the reconstructed sequence. As a result, aligning the reconstructed sequence back to the genome can reveal large gaps, often with lengths that are multiples of the monomer size, corresponding to these unrepresented variants. Therefore, improving and expanding the monomer library to better capture centromere sequence diversity is an important direction for reducing such gaps in future analyses.”
